# Use of Topical Negative Pressure in British Servicemen With Combat Wounds

**Published:** 2011-08-19

**Authors:** Jowan G. Penn-Barwell, C. Anton Fries, Lesley Street, Steven Jeffery

**Affiliations:** ^a^Academic Department of Military Surgery and Trauma, Royal Centre for Defence Medicine (RCDM), ICT Research Park, Vincent Drive, Edgbaston, Birmingham B15 2SQ, United Kingdom; ^b^Queen Elizabeth Hospital Birmingham, Mindelsohn Way, Birmingham B15 2WB, United Kingdom

## Abstract

**Objective:** The objective of this study was to characterize the use of topical negative pressure therapy in combat wounds. **Methods:** This study was a retrospective review of the records of patients whose wounds were managed with topical negative pressure between April 2007 and March 2008. The main outcome measure was episodes of antibiotic prescription, which was used as a surrogate marker of clinically relevant infection. **Results:** Of the 62 cases identified, 25 clinical notes were unavailable and were excluded from the study leaving 37 included cases. All but one of the cases was male with an average age of 29 (19-39) and New Injury Severity Score (NISS) of 21.3 (14.4-28.1). In 20 cases, topical negative pressure was changed less than once per 4.9 days on average, and in the remaining 17 cases, this was done more frequently. Comparison of the rate of antibiotic prescription between these groups reveals a significantly higher rate in the cohort managed with more frequent topical negative pressure changes. However this relationship was not borne out in a multiple variable analysis. **Conclusion:** This study describes the use of topical negative pressure in the management of a uniquely challenging group of patients. Statistical analysis of relatively small numbers is challenging but these results support the current complex wound management strategies where wounds are temporized with topical negative pressure for several days following thorough wound debridement. This period allows patients to be physiologically stabilized, other injuries to be addressed and appears not to be associated with increased infections.

The Royal Centre for Defence Medicine (RCDM) is based in South Birmingham and is the receiving facility for casualties evacuated from Iraq and Afghanistan. Since the start of hostilities in Afghanistan in 2001, the unit has gained considerable experience with managing complex combat extremity trauma (see Fig 1).

The principles of managing high-energy combat wounds has changed little over the last half-century of conflict and remains early debridement and irrigation and delayed closure. One of the main differences in the management of combat wounds from current conflicts compared with those from the previous half-century has been the use of topical negative pressure (TNP). Over the last 10 years, the use of TNP has proved to be a useful adjunct in managing high-energy traumatic wounds management^1^^,^^2^ and the use of TNP in the treatment of combat wounds is now standard practice in the US and UK military medical services. This has made a significant impact on the management of amputees and has facilitated the development of the technique of “stump salvage” where multiple procedures including serial debridement, reconstruction, and temporizing with TNP to maintain maximum stump length.^3^

As the familiarity with this technique has developed, the way it has been employed has also evolved. The intention of this retrospective review was to systematically examine the use of TNP at RCDM to allow comparison of our experience with those of our international military colleagues.

## PATIENTS AND METHODS

Military patients admitted to RCDM between April 2007 and March 2008 that were treated with TNP were identified from the log kept by the TNP specialist nursing team. The clinical notes and electronic prescribing database were examined and cross-checked against the Joint Theatre Trauma Registry compiled prospectively by the Academic Department of Military Emergency Medicine. Cases were excluded if there were clinical records inadequate or unavailable. These 3 data sources were examined for the information listed in Table 1. The treatment and outcome variables are shown in Table 2. The delay between injury and first application of TNP is the total delay between injury and first TNP use, including time in field hospitals when TNP was not available. It is important to note that dressing changes in theaters were often part of other operations and may not have been regarded as necessary otherwise, and hence the number of dressing changes per patient may be artificially inflated. Antibiotic use was used as a surrogate marker of clinically significant infection and was therefore measured in an episodic fashion, that is, if 2 separate antibiotics were prescribed simultaneously, that was taken as 1 episode. The total duration of inpatient care was measured as time from injury to discharge from RCDM. It should be noted that many patients were discharged to the care of the Defence Rehabilitation facility with medical and nursing staff who were increasingly able to manage patients' more complex needs than would be appropriate for traditional community care.

Analysis was performed in 2 ways. First, cases were divided into those with frequent TNP changes (once per 4.9 days or less) and those with infrequent TNP changes (every 5 days or more). These two groups were compared with respect to antibiotic episodes using a student *t* test.

The second technique was a multiple regression analysis, which examined whether the different ways TNP had been used in the cases was related with the principle outcome measure of antibiotics episodes. This analysis was performed by a specialist bio-statistician using PASW Statistics 18 (SPSS Inc, Chicago, Illinois).

## RESULTS

Of the 62 cases identified, 25 clinical notes were unavailable, leaving 37 included cases that form the basis of this study. For the calculation of duration of inpatient care, one case was excluded from analysis, as he remained an inpatient for a prolonged period due to his immigration status and lack of appropriate facility to discharge him to.

The characteristics of the patient population are shown in Table 2, with the age and gender bias being typical of a military population. The mean New Injury Severity Score (NISS) of 21.3 and the mean critical care stay of 4.6 days give a measure of the severity and complexity of these patient's injuries.

**Figure 2:**
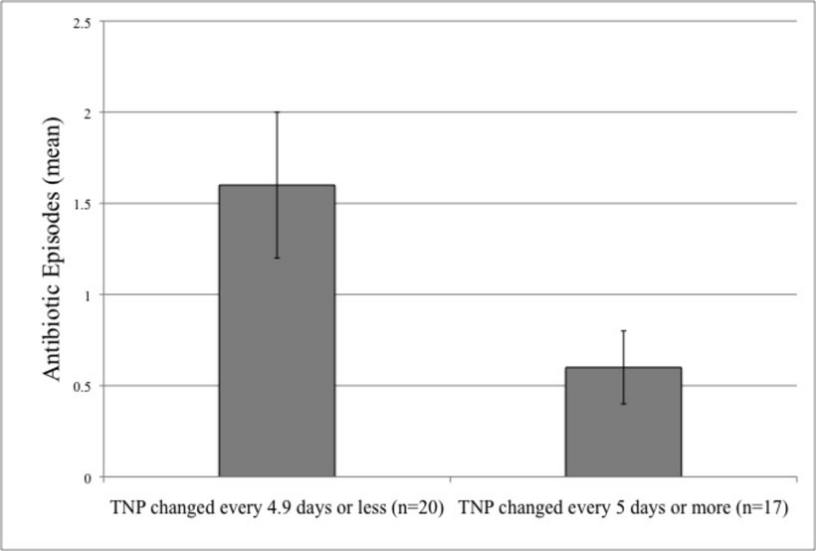
Antibiotic episodes by TNP change frequency.

Twenty cases had TNP changed every 4.9 days or less, while 17 cases were changed less frequently. The frequencies of antibiotic episodes in these 2 groups are shown in Figure 2, which are significantly distinct (*P* = .017). The multiple regression analysis revealed a negative correlation between the delay to first use of TNP and episodes of antibiotic prescription during TNP use with a significance of *P* = .018, though this significance decreased to *P* = .038 once injury severity was controlled for. There was no relationship between frequency of TNP dressing changes and infection rate.

## DISCUSSION

This study describes the use of TNP in the management of a uniquely challenging group of patients. Analysis of relatively small numbers is problematic, but these findings appear to suggest that less frequent changes of TNP are not associated with increased wound infections.

Patients with combat injuries frequently have competing surgical priorities of massive soft tissue loss, open fractures, torso injuries, physiological derangement, and sepsis. In these cases, the use of TNP is an attractive option for temporizing wounds while fractures are stabilized, infections treated, and patients become stable enough for prolonged reconstructive surgery. While TNP has become ubiquitous in the UK/US Defence Medical Services in patients with significant combat wounds,^4^^,^^5^ there is a lack of high-level evidence supporting this.^6^^,^^7^ Such patients are challenging to include in clinical trials, nor are these patterns of injury commonplace in civilian practice.

In their 2008 systematic review of the literature, Ubbink et al^8^ concluded that TNP's superiority over traditional dressings was not proven. Since then, Stannard et al^9^ have published the results of the only randomized study comparing TNP to standard dressings for managing open fractures. Over five years, 59 patients with open fractures that could not be closed primarily were randomized to receive either standard dressings or TNP. They found significantly lower rates of infection in the TNP equating to a fifth of the relative risk.^9^

There have been 2 case series examining TNP use with combat wounds, both published by the US military. In both the acute, that is, within 24 hours of wounding^10^ and delayed phase, that is, following repatriation to the United States,^11^ these studies found improved rates of healing, reduced levels of infection, and improved qualitative status of the wounds involved when compared to historical experiences.

**Figure 3:**
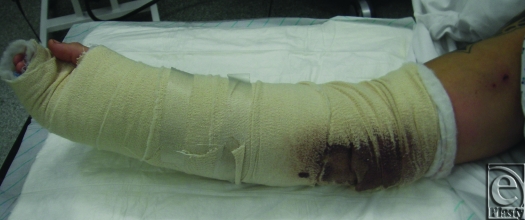
12-hour old dressing saturated by wound exudate.

The lack of correlation between time of first application and any of the outcome markers does not support the argument that TNP should be applied at the time of the first surgical intervention. However, the clinical experience has been that the copious exudate produced by these wounds over the leads to the saturation of traditional dressings in the prolonged transport times between the field hospitals and the United Kingdom. Topical negative pressure avoids patient's wounds remaining in contact with soiled dressings for many hours, which, aside from infection risk, many patients report as distressing due to the odor of saturated dressings (see Fig 3).

Analysis of these data show that delay between TNP dressing changes in this series does not relate to rate of infection. This research may therefore support leaving TNP dressings in situ for longer than the 48 hour standard of care in the civilian setting.^12^ This finding is of great importance and adds to the limited amount of evidence relating to optimum timings of dressing changes in military wounds, whether using TNP or conventional dressings.

**Figure 4:**
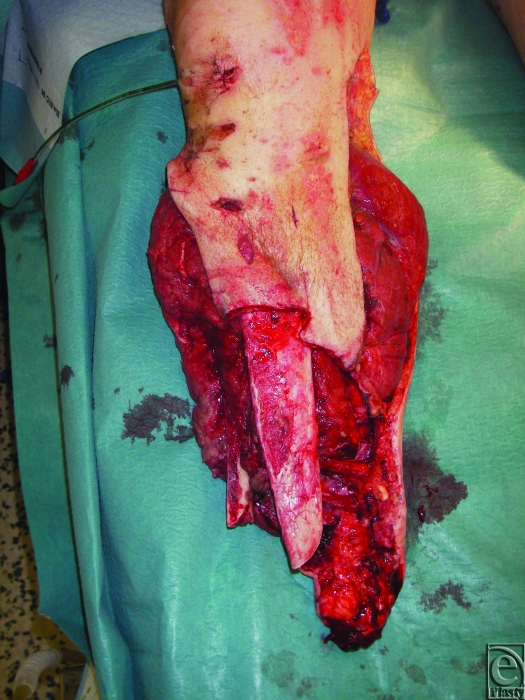
Traumatic amputation of lower extremity.

**Figure 5:**
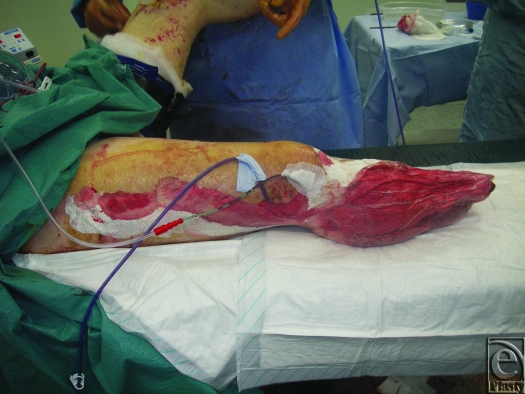
Same leg temporized with TNP.

**Figure 6:**
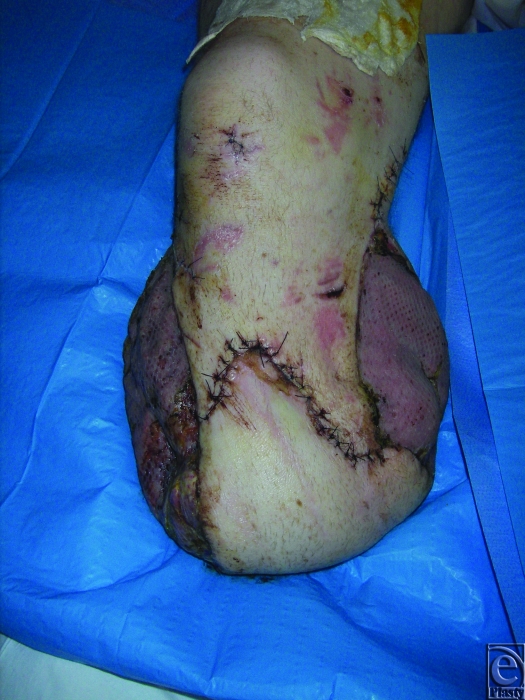
Maximum ‘stump-salvage’ achieved with a variety of closure techniques.

Current practice in RCDM is largely informed by the clinical experience that sufficiently debrided wounds can be safely temporized with unchanged TNP for several days; indeed 3 patients in this series had their dressings unchanged for 8 days with no adverse consequences. This allows for the patient to have other clinical priorities addressed or physiologically normalized to the point that they can tolerate the lengthy surgery associated with definitive skeletal stabilization and complex reconstructive plastic surgery—see Figures 4, 5, and 6, which show a typical injury and treatment progress. At RCDM, patients whose wounds are temporized with TNP for several days are returned to theater urgently if they exhibit any physiological signs of wound sepsis. Both sponge- and gauze-based TNP systems were used in these patients and were subjectively judged to be comparable.

As a qualitative description of a limited case series, these data must be interpreted in the context of the existing literature. The unavailability of 40% of patient notes is potentially significant source of bias, though no systematic factors influencing note availability was obvious. The use of antibiotic prescription as a surrogate marker for wound infection has limitations and is clearly likely to have inflated the rate of “infections” in our study, including both the wound infections of interest and others. The use of multivariant regression analysis is not regarded as a very robust study tool, though it can, as it has in this case, be used to examine the assumptions on which clinical decisions are based.

## CONCLUSIONS

Topical negative pressure is just one of a number of techniques for managing military high-energy injuries and should not be seen as an alternative to the established principles of early debridement, irrigation, fracture stabilization, and delayed closure. A deviation from the civilian standard of TNP dressing changes every 48 hours is supported by this research. Table 3.

## Acknowledgments

Dr Sanchia Goonewardene, Research Fellow, Worcester Acute Hospitals NHS Trust, is thanked for her help with data collection. Joint Theatre Trauma Registry, The Academic Department of Military Emergency Medicine, is thanked for collecting, collating, and identifying the appropriate data for this article. The authors also thank Peter Nightingale, Statistician, University Hospitals Birmingham NHS Trust, UK.

## Figures and Tables

**Figure 1:**
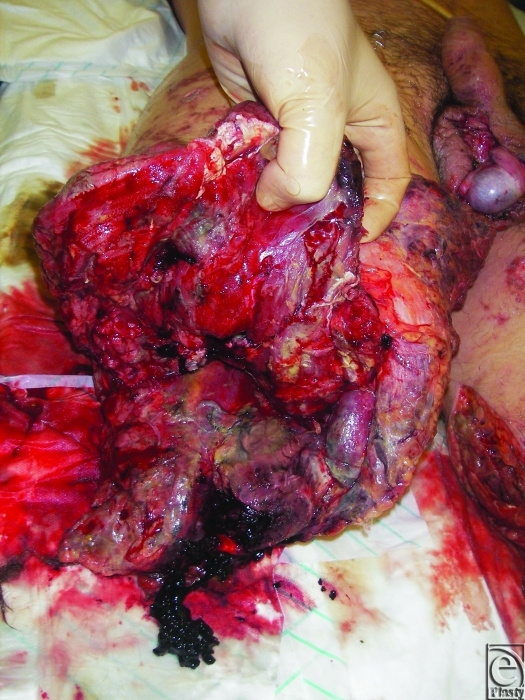
Typical high-energy combat trauma with extensive soft tissue damage.

**Table 1:** Data collected from clinical records

Patient factors	Patient demographics
	Mechanism and site of Injury
	New Injury Severity Score (NISS)
Descriptors of management	Number of debridements
	Duration of critical care
	Duration of secondary care
Variables	Delay between injury and TNP first use
	Frequency of TNP dressing changes
	Duration of TNP use
	Debridements during TNP
Outcome Measure	Infections requiring new prescription of antibiotics

**Table 2:** Patient characteristics*

Age	29 (19-39)
Sex (M:F)	36:1
Blast injury (%)	26/37 (70%)
Gun-shot wound (%)	11/37 (30%)
NISS (95% CI)	21.3 (14.4-28.1)
Critical Care Days (95% CI)	4.5 (3.1-5.9)

*CI indicates confidence interval; NISS, New Injury Severity Score.

**Table 3:** Variables and main outcome measures

	Mean	95% CI
Delay between injury and TNP in days	5	3-6
Duration of TNP in days	15.5	10.5-20.5
TNP changes on ward	2.8	1.9-3.7
TNP changes in Operation theater	1.1	0.8-1.5
Frequency of TNP dressing changes in days	4.7	3.2-6.2
Antibiotic prescription episodes	1.1	0.8-1.5
